# Cancer-associated mesenchymal stem/stromal cells: role in progression and potential targets for therapeutic approaches

**DOI:** 10.3389/fimmu.2023.1280601

**Published:** 2023-11-03

**Authors:** Ali Hazrati, Kosar Malekpour, Zahra Mirsanei, Arezou Khosrojerdi, Nasim Rahmani-Kukia, Neda Heidari, Ardeshir Abbasi, Sara Soudi

**Affiliations:** ^1^ Department of Immunology, School of Medicine, Tehran University of Medical Sciences, Tehran, Iran; ^2^ Department of Immunology, School of Medicine, Iran University of Medical Sciences, Tehran, Iran; ^3^ Department of Immunology, School of Medicine, Shahid Beheshti University of Medical Sciences, Tehran, Iran; ^4^ Infectious Diseases Research Center, Birjand University of Medical Sciences, Birjand, Iran; ^5^ Department of Biochemistry, School of Medicine, Shiraz University of Medical Sciences, Shiraz, Iran; ^6^ Department of Immunology, Faculty of Medical Sciences, Tarbiat Modares University, Tehran, Iran

**Keywords:** MSCs, cancer, immunosuppression, metastasis, tumor growth, angiogenesis

## Abstract

Malignancies contain a relatively small number of Mesenchymal stem/stromal cells (MSCs), constituting a crucial tumor microenvironment (TME) component. These cells comprise approximately 0.01–5% of the total TME cell population. MSC differentiation potential and their interaction with the tumor environment enable these cells to affect tumor cells’ growth, immune evasion, metastasis, drug resistance, and angiogenesis. This type of MSC, known as cancer-associated mesenchymal stem/stromal cells (CA-MSCs (interacts with tumor/non-tumor cells in the TME and affects their function by producing cytokines, chemokines, and various growth factors to facilitate tumor cell migration, survival, proliferation, and tumor progression. Considering that the effect of different cells on each other in the TME is a multi-faceted relationship, it is essential to discover the role of these relationships for targeting in tumor therapy. Due to the immunomodulatory role and the tissue repair characteristic of MSCs, these cells can help tumor growth from different aspects. CA-MSCs indirectly suppress antitumor immune response through several mechanisms, including decreasing dendritic cells (DCs) antigen presentation potential, disrupting natural killer (NK) cell differentiation, inducing immunoinhibitory subsets like tumor-associated macrophages (TAMs) and Treg cells, and immune checkpoint expression to reduce effector T cell antitumor responses. Therefore, if these cells can be targeted for treatment so that their population decreases, we can hope for the treatment and improvement of the tumor conditions. Also, various studies show that CA-MSCs in the TME can affect other vital aspects of a tumor, including cell proliferation, drug resistance, angiogenesis, and tumor cell invasion and metastasis. In this review article, we will discuss in detail some of the mechanisms by which CA-MSCs suppress the innate and adaptive immune systems and other mechanisms related to tumor progression.

## 1 Introduction

Tumors are formed by complex environmental components, including various cells such as fibroblasts, mesenchymal stromal/stem cells (MSCs), endothelial cells, immune cells, and factors involved in intercellular communication, including extracellular vesicles (EVs), cytokines, and extracellular matrix (1). These components together and coordinate with each other to form the tumor microenvironment (TME) that helps tumor growth. Also, these cells, in relation to the environment, affect the physicochemical conditions of the tumor site, including fibrosis, hypoxia, extracellular pH, and increased interstitial fluid pressure, and in this way, they contribute to tumor growth (2). Tumor-initiating cells (TICs), also known as cancer stem cells (CSCs) (3), are subpopulations of tumor cells in TME that can start tumors and trigger relapses (4, 5). It is believed that CSCs originate from differentiated cells that have undergone mutations or from stem cells that are resident in adult tissues (6–8). Several biomarkers are used to identify CSCs and have been correlated with diagnosis, therapy, and prognosis (9). Despite having specific biomarkers, CSCs are regarded as highly plastic, leading to changes in their phenotype and function over time due to this plasticity (6). It is known that CSCs are capable of forming their microenvironment in favor of tumor growth through the recruitment and activation of specific cell types, including MSCs, which are referred to as cancer-associated mesenchymal stromal/stem cells (CA-MSCs) (9, 10). MSCs usually exist in various mesenchymal tissues such as bone marrow, adipose tissue, cartilage, dental pulp, umbilical cord, and umbilical cord blood, and they can be isolated from these tissues (11). These cells have a high ability of self-renewal and differentiation and can differentiate into a variety of different types, including osteocytic, adipocytic, and chondrocytes. Also, due to the increased expression of chemokine receptors related to inflammation, these cells can migrate to the site of inflammation and induce their actions there. One type of chronic inflammatory site in the body of patients is tumor tissue. Therefore, MSCs can migrate to the tumor tissue and perform various actions there under the influence of the tumor environment (12). MSCs can play their role by producing multiple cytokines, growth factors, and extracellular vesicles, including exosomes (13, 14). MSCs and their exosomes play a role in the treatment of many diseases, such as orthopedic diseases (15), inflammatory diseases, infectious diseases (16, 17), etc. However, it seems that the presence of these cells (CA-MSCs) in tumor tissue can lead to tumor progression.

The CA-MSCs have the potential to modify the stroma and establish an optimal microenvironment for the restoration of CSCs and the progression of tumors. Crosstalk between cancer cells and MSCs within a microenvironment has also altered the CSC phenotype in different cancers. The current evidence suggests that the primary source of CA-MSCs employed by cancer cells derives from both distantly recruited MSCs and resident MSCs, a principal origin of the cells (6, 18, 19). It is typical for tumor and non-tumor cells in the TME to influence the phenotypical and functional transition of naive MSCs into CA-MSCs (20). Multiple mechanisms are involved in crosstalk between CSCs/cancer cells and CA-MSCs, including cell-to-cell interactions (21), secretion of exosomes (22), and paracrine secretion of inflammatory mediators, cytokines, and growth factors (23–25). Considering CA-MSCs and their mediators that play a unique role in the TME, more studies are required to elucidate how CA-MSCs/CSCs interact to overcome tumor immunity.

## 2 Regulation of immune responses

It has been observed that CA-MSCs interact with tumor cells and recruit various immune cells, especially macrophages and neutrophils, as well as myeloid-derived suppressor cells (MDSCs), then skew their phenotype in favor of tumor cells, immune response suppression, and tumor development, as it is summarized in **Table 1**.

**Table 1:** The mechanisms of CA-MSCs in innate immune response suppression.

Cell Type	Mechanism	Example	Effect	Reference
**Macrophage**	Decrease pro-inflammatory molecules	TNF-α, IP10, RANTES, MIP-1	Switch M1 to M2 phenotype	(26)
	Increase anti-inflammatory molecules	TGF-β IL-10	Switch M1 to M2 phenotype	(27–29)
	Increase monocyte migration mediators	MCP-1, SDF-1, Chi3L1, M-CSF1, IL-6, IL-8	Increase migration of monocyte and M2-Macrophage to TME	(30–33)
	Expression of Chemokine-Chemokine Receptor	CCR2 (Induced by HIF-1 or TNF-α, binds to CCL2, CCL7, CCL12)	Increase migration of monocyte and M2-Macrophage to TME	(34, 35)
		CX3CL1	Increase migration of monocyte and M2-Macrophage to TME	(36)
		CXCL10 (binds to CXCR3, increase CXCL16 secretion)	Increase migration of MSCs to TME	(37)
		CCL5 (binds to CCR5, increase SCF-1 secretion)	Induces macrophages and MDSCs migration to TME	(37)
**Neutrophil**	Expression of Chemokine-Chemokine Receptor	CXCR2 (binds to CXCL1, CXCL2, and CXCL5)	Increase in neutrophil recruitment to TME	(38)
		CLCF1 (Increasing CXCL6 and TGF-β expression)	Induces the polarization of N2-phenotype neutrophils in TME	(39)
	Increase neutrophil activation pathway	IL-6-mediated STAT3-ERK1/2 axis, SDF-1α	Induce the chemotaxis and activation of neutrophils	(40, 41)
**Natural killer cell**	Inhibition of NK cell activation	PGE2 IDO Suppression of stimulatory receptors (NKP30,44)	Induction of inactivity and unresponsive state	(42, 43)
		Reduction of MIC-A, B (ligands of NK-activating receptors) in tumor cell	Decrease the cytotoxicity and γ-IFN secretion	(44)
	Generate anti-inflammatory molecules	TGF-β	downregulates NKG2D, NKp30, NKp46, DNAM-1 expression	(45, 46)
**Dendritic cell**	Induction of rDCs	IL6-Stat3 pathway activation	recruited and transdifferentiated normal DC into rDCs	(47)
		Production of VEGF	Inhibition of NF-kB Upregulation of PD-L1 expression	(48–50)
**Myeloid-derived suppressor cells**		CCL2 (CCL2-STAT3 signaling enhances the recruitment of MDSC to TME.)	Inhibition of anti-tumor response promoted tumor growth	(51)

### 2.1 Regulation of innate immune responses

#### 2.1.1 Regulation of macrophage functions

Tumor-associated macrophages (TAMs) are divided into two distinct subsets, M1, stimulated by lipopolysaccharide (LPS) alone or combined with Th1 cytokines, and M2, activated by Th2-related cytokines. Producing tumor necrosis factor (TNF), reactive oxygen species (ROS), and facilitating antibody-dependent cellular cytotoxicity, M1-type macrophages have an anticancer function in the TME (52). While extracellular matrix (ECM) remodeling, tumor angiogenesis, immune suppression, and metastasis are all factors that M2-type macrophages use to promote tumor growth (53).

CA-MSCs can also switch macrophages from a pro-inflammatory M1 phenotype to an anti-inflammatory M2 phenotype, which enhances macrophage immunosuppressive effect. For instance, the results of a study showed lower production levels of pro-inflammatory cytokines, such as TNF-α, IP10 (IFN-γ inducible Protein 10kDa), RANTES, and macrophage inflammatory protein-1 alpha (MIP-1α), when macrophages were co-cultured with CA-MSCs derived from gastric cancer compared to normal MSCs (26). The result of *in vitro* studies have provided more detailed insight into the role of the interaction between MSCs and macrophages in the development of tumors and their metastasis. Researchers have discovered that CA-MSCs express high levels of CCR2, which binds to CCL2, CCL7, and CCL12 (34). CCR2 is also highly expressed in macrophages and regulates myeloid cell recruitment to tumor sites (35). After macrophages were specifically eradicated from melanoma, lymphoma, and breast cancer, Ren G et al. found that the tumor-promoting function of CA-MSC cells was also abolished once macrophages were specifically eliminated from these cancers (34). It is also accompanied by evidence that the tumor-promoting properties of CA-MSCs have been attenuated in CCR2-deficient mice (34, 54).

Additionally, Cascio S’s research on CA-MSCs in ovarian cancer has declared that these cells, which express CX3CL1, CCL2, and TGF-β, lead to the migration of CCR2^+^ monocytes and M2 TAMs to the TME and the complete elimination of responses to immune checkpoint inhibitor therapy (36). TNF-α and the TME hypoxic condition stimulate MSCs to express CCR2, followed by the production of CCL2, CCL7, and CCL12, and macrophage recruitment (34, 37). According to a comprehensive study on the role of MSCs and macrophages in breast cancer, a triple communication between tumor cells, macrophages, and MSCs contributed to the progression of breast cancer by interacting with chemokines and chemokine receptors. This interaction is conducted by two signaling loops regulated by the hypoxia-induced factor-1α (HIF-1α) (37). A signaling loop occurs when hypoxic CA-MSCs secrete CXCL10, which binds to the CXCR3 of hypoxic breast cancer cells and results in CXCL16 secretion (37). Upon binding to CXCR6 on hypoxic CA-MSCs, CXCL16 promotes the expression of CXCL10, resulting in further MSC recruitment to the tumor site due to more CXCL16 expression (37). Following this, another signaling loop is activated as well: CA-MSCs secrete CCL5, which binds to CCR5 on the surface of cancer cells and triggers the expression of the chemokine colony stimulating factor-1 (CSF-1) on those cancer cells, which then induces macrophages and MDSCs migration (37). Further investigation revealed that not only are CSF-1 and CCR5 targets of HIF-1α, but human samples confirmed that these loops exist as well (37).

#### 2.1.2 Regulation of neutrophils functions

TANs, or tumor-associated neutrophils, phenotypically are diverse and have various functions (55, 56). These cells are an essential component of the TME. Neutrophils, like TAMs (M1 and M2), can be anti-tumorigenic (N1) or pro-tumorigenic (N2), depending on whether they have been activated by TGF-β (57, 58). However, unlike TAMs, the distinction between TAN N1 and N2 phenotypes is made by the activation level as opposed to by various polarizing chemicals (59). CA-MSCs also alter neutrophil functions in favor of tumor growth. For example, when CA-MSCs are stimulated by TNF-α in a mouse breast cancer model with the 4T1 cell line, lung metastasis is increased (38). This phenomenon can be attributed to increased neutrophil recruitment to tumor primary sites. CXCR2 ligands, including CXCL5, CXCL2, and CXCL1, produced by TNF-α activated MSCs account for the deposition of neutrophils at tumor primary sites and contribute to the metastatic microenvironment formation (38).

According to Zhu et al., gastric cancer-derived MSCs (GC-MSCs) interact in 2 directions with neutrophils (41). Firstly, by STAT3-ERK1/2 IL-6-mediated axis, GC-MSCs can upregulate the neutrophil’s activation and chemotaxis, resulting in their survival. The second function of activated TANs is facilitating the differentiation of MSCs into cancer-associated fibroblasts (CAFs) (39).

#### 2.1.3 Regulation of natural killer cell functions

NK cells naturally respond to tumor cells (60, 61), but NK cells’ activity is determined by the engagement of their stimulatory or inhibitory receptor (62). The NK cell receptors include NK group 2D (NKG2D), DNAX accessory molecule 1 (DNAM-1), NKp46, NKp44, and NKp30, which serve as stimulatory receptors. In contrast, killer immunoglobulin-like receptors (KIRs) and CD94/NK group 2A (NKG2A) are inhibitory. TMEs in solid tumors contain different cells and soluble inhibitory factors, such as MSCs, which impair the function of NK cells infiltrating the tumor (63, 64). TGF-β is crucial in tumors’ CA-MSC and NK cell interactions. Evidence suggests that CA-MSCs secreted TGF-β inhibits IFN-γ production and NKG2D activation on cell surfaces. For example, through miR-183 activation, TGF-β inhibits DAP12 transcription and NKp30 and NKG2D expression, effectively suppressing NK cells (45). Additionally, TGF-β, after activating the SMAD2/3 pathway, downregulates NKG2D, DNAM-1, NKp30, and NKp46 expression in the *in vitro* condition (46).

#### 2.1.4 Regulation of dendritic cells functions

Dendritic cells that infiltrated to tumor tissue (TiDCs), as a heterogeneous group of DCs, express a high level of MHC class I and class II complexes, costimulatory and adhesion-related molecules. These cells are essential for initiating and controlling innate and acquired (or adaptive) immune responses (43). According to recent research, CA-MSCs can suppress the processing and presentation of antigens by DCs and suppress the adaptive immune response (naïve T cell activation), which can help cancer cells evade the immune system (65). As a result of IL-6-mediated STAT3 pathway activation, normal DCs are recruited and transdifferentiated into regulatory DCs (rDCs), which have no antigen presentation but release suppressive mediators, including IDO (47). These mediators (such as IDO) limit T cell-mediated immunity by inducing T cell anergy and Treg cell proliferation (66).

#### 2.1.5 Regulation of mast cells

Mast cells (MCs) are corporate in both tumor progression and suppression, depending on the cancer type, MCs localization, and degree of tumor progression (67–69). One aspect of MCs’ function as cancer promoters is to stimulate angiogenesis, lymphangiogenesis, and degradation of ECM by releasing a wide range of pro-angiogenic molecules [vascular endothelial growth factors (VEGFs), histamine, heparin, and stem cell factor (SCF)] (70–74), lymphangiogenic molecules (75), proteases, and matrix metalloproteinase-9 (MMP-9) (76–78). Meanwhile, MCs increase antitumor inflammation, induce tumor cell apoptosis, and reduce cancer cell invasion and metastasis due to their antitumor effector production, including TNF-α, chondroitin sulfate, tryptase, and IL-1 (69). CA-MSC immunosuppressive effects through MCs have received only minimal comprehensive research so far. Researchers recently found that MCs and CAFs contributed to the morphological transformation of benign epithelial cells into cancerous ones in a prostate cancer micro-tissue model (79).

### 2.2 Regulation of adaptive immune response

T lymphocytes, such as cytotoxic T lymphocytes (CTLs), Treg cells, and effector helper T (Th) cells, are the prominent soldiers of the immune system in driving and modulating adaptive immune responses (80). There is mounting evidence to support that CA-MSCs modulate T-cell activities and functions (**Table 2**).

**Table 2:** The mechanisms of CA-MSCs in adaptive immune response suppression.

Cell Type	Mechanism	Example of Responsible Mediators	Effect	Reference
**T helper lymphocytes**	Differentiation into immunoinhibitory subpopulation	TGF-β1	Shift to Th17 differentiation	(38, 81)
		TSLP	Shift to Th2 differentiation	(82)
**Cytotoxic T lymphocytes**	Immune tumor exclusions	Removing TCD8+ cells surrounding tumors	Resistance to cancer immunotherapy	(36)
		Release VEGFs (lead to decrease cell adhesion molecules ICAM-1/2, VCAM-1)	Decrease CD8+T cells recruitment to tumor site	(83)
		IL6	Decrease CD8^+^T cells infiltration	(84)
		Releasing CXCL12 (facilitate CTLs trafficking away from juxtatumor area)	Decrease the frequency of infiltrating CTLs in tumor islets	(83, 85–87)
	CTL activity suppression	TGF-β	Inhibition of Cytotoxicity activity	(88)
		βig-h3 (binds to CD61, inhibition of Lck pathway)	Decreases in TCR signaling transduction	(89)
***Regulatory T lymphocytes***	Stimulate Treg cells’ migration	CXCL12 (binds to CXCR4)	Increase the frequency of infiltrating Treg in TME	(90)
		CCL5	Regulatory T cells stimulated through RANKL-RANK signaling	(91, 92)
	Induction and maintenance of Treg cell	TGF-β (induce Foxp3 expression in T lymphocytes)	Differentiation of naive T cells into CD4 + CD25 + Treg cells.	(93)
		Expression of CD73, DPP4, and B7H3	Transform CD4 + T cells into Foxp3+ Treg cells	(94)

#### 2.2.1 Regulation of T lymphocytes

There are five major types of Th cells: Th22, Th17, Th9, Th2, and Th1 (95), all derived from naive T CD4^+^ cells. Th1 and Th2 cells, involved in cellular and humoral immunity, respectively, by releasing various particular cytokines (96). Different studies showed that MSCs can decrease the differentiation of naïve T cells into inflammatory subsets, including Th1 and Th17 (11). MSCs that are recruited to the tumor site have an essential role in modulating the responses of T cells. The results of studies show that CA-MSCs, by producing TGF-β, can lead to the induction of regulatory T cell differentiation from naïve T cells (97). Also, they increase the differentiation of IL10-producing Tr1 cells by producing prostaglandine E2 (PGE2) (98). This result was demonstrated in mice infected with *Helicobacter pylori* and receiving BM-MSCs 10 months after infection. It was shown that this treatment skews the immune response to an immunosuppressive state by inducing Tr1 cells and Treg cells, possibly contributing to an immune microenvironment that tolerates *H. pylori*-mediated gastric cancer progression. *In vitro* studies also suggested that Treg cells induced by TGF-β suppress the cytolytic potency of CD8^+^ T cells and NK cells against T47D breast cancer (99). Also, as mentioned in the previous sections, CA-MSCs can affect the TCD4^+^ cells’ function by affecting innate immune cells such as DCs and macrophages (100).

CTLs, or CD8^+^ T cells, mediate cytotoxic activities by inducing tumor cell apoptosis (101, 102). Several inflammatory cytokines secreted by effector T cells, such as IFN-γ and TNF-α, stimulate CA-MSCs to produce TGF-β, CXC chemokines such as CXCL10, CXCL9, and CXCL11, as well as the large amounts of inducing nitric oxide synthetase (iNOS) (103) and IDO (104). All of the mentioned substants suppress effector T lymphocytes in humans, including TCD4^+^ and CTLs. As Sandra Cascio demonstrates, CA-MSCs have a role by removing TCD8^+^ cells surrounding tumors and creating “immune tumor exclusions” that prevent TCD8^+^ cells from interacting with cancer cells, causing resistance to immune checkpoint inhibitor (ICIs) cancer therapy (36). It has been discovered in another study that the injection of B16 melanoma cells into allogeneic mice can result in tumor formation only when it is combined with BM-MSCs (105). In addition, CA-MSCs derived from cervical cancer patients were also shown to prevent antigen-specific CD8^+^ T lymphocytes from destroying CaSki cells *in vitro*, a human cervical cancer cell line (106).

#### 2.2.2 Regulation of B lymphocyte functions

**Figure 1:**
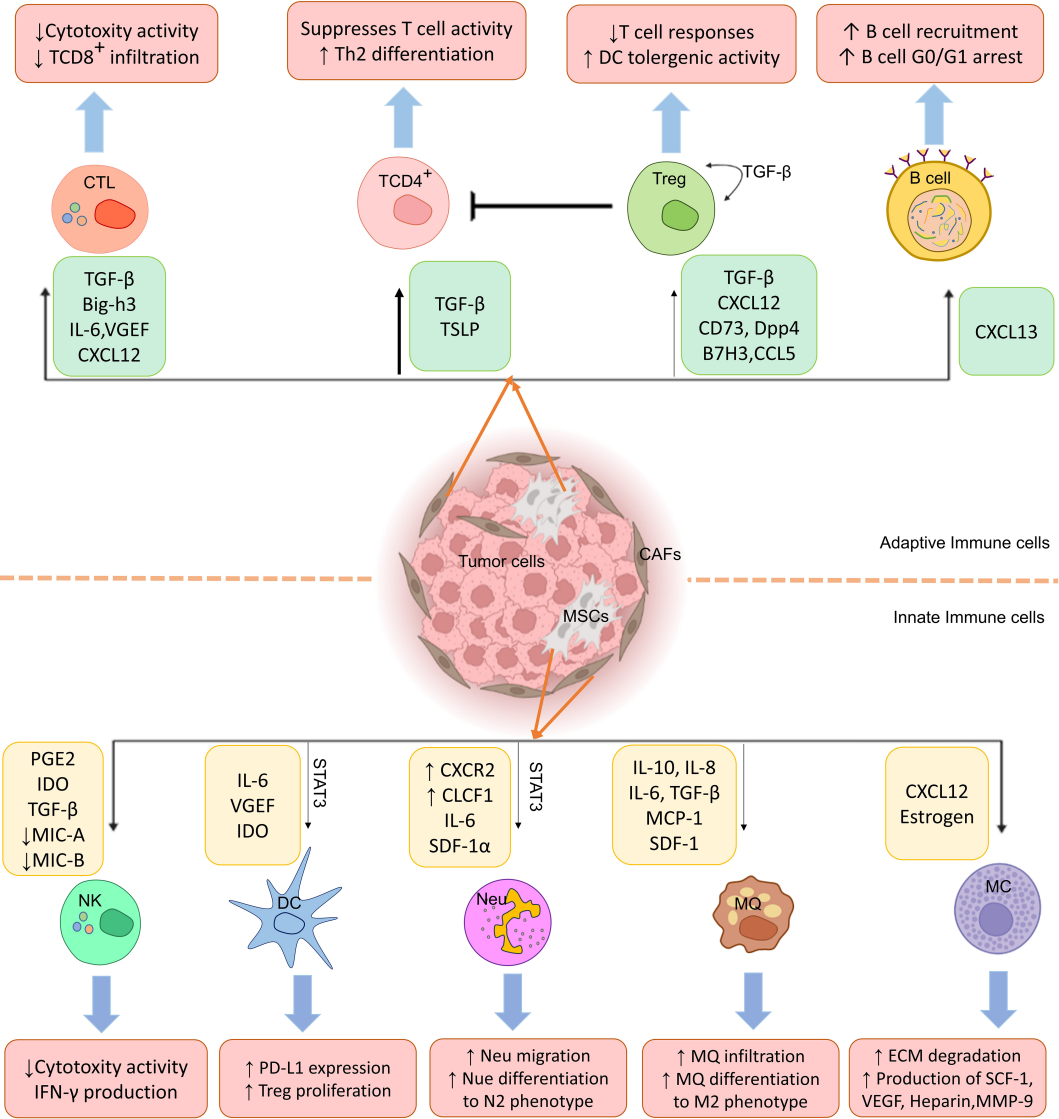
Cancer-associated mesenchymal stem cells (CA-MSCs) affect innate and adaptive immune system cells. As shown in the figure, MSCs disrupt immune system cells’ functions in the tumor environment by producing various mediators and leading immune responses to the expansion of M2 macrophages, regulatory T cells, and N2 neutrophils and suppression of CTL and NK cell responses.

A tumor immune microenvironment also contains B cells that can act as potentiators or inhibitors of antitumor immunity and regulate cancer progression (107). A lot of study has been shown recently on how CA-MSCs affect T lymphocytes, but very few studies have looked at how MSCs affect B cells. However, there is evidence that BM-MSCs can cause B cells to enter the G0/G1 phase of the cell cycle and decrease B cell growth and antibody synthesis (108, 109). Despite these results, more study is required to comprehend the consequences of B cell and MSC interaction during tumor formation. According to current studies, CXCL13 is the only chemokine secreted by CAFs that enhances B cell recruitment (110). Therefore, the interaction of CA-MSCs and CAFs with B lymphocytes to suppress antitumor responses needs further investigation (**Figure 1**).

## 3 Role of CA-MSCs in promoting cancer growth

In the last decade, many studies have been conducted that have shown that MSCs cause the growth and proliferation of tumor cells through the effect on signaling pathways (111). From another aspect, it has been shown that these cells can impede the growth of cancer cells (112), so this issue is being discussed. Interestingly, the anti-tumor or tumor growth-supporting effects of MSCs depend on the source and type of MSCs, so BM-MSCs and adipose tissue derived MSCs (AT-MSCs) have the capacity to promote tumor growth, but UCB-MSCs inhibit tumor growth (113, 114). MSCs increase the growth and malignancy of cancer cells in several different ways, including 1) production of cytokines and chemokines. 2) Phenotypic and metabolic characteristics of MSCs. 3) Immune cell modulation and immunosuppressive effects. 4) Effect on TME. 5) Effect on non-coding RNAs such as miRNAs. In the following, we briefly describe these factors. Studies supporting the function of MSCs to favor tumor growth are summarized in **Table 3**.

**Table 3:** The role of CA-MSCs in increasing tumor growth by different mechanisms.

MSC origin	Cancer types	Mechanisms	Outcomes	ref
GC-MSCs	Gastric cancer cells	M2 macrophage polarization by IL-6/IL-8/JAK2/STAT3 pathways	Promotes growth, metastasis, and EMT in gastric cancer	(26)
BM-MSC	Melanoma	Decrease of T cell proliferation by IL-10-STAT3	Promotes growth of melanoma	(65)
BM-MSC	Breast cancer	Induction of Treg by secreted TGF-β	Increased growth of breast cancer	(97)
A-MSC	Ovarian cancer	Inhibit function of CD8^+^ T by expression CCL2, TGFβ1 CX3CL1،	Promote tumor growth	(36)
MSCs TA-	Lung cancer	Inhibit NK cells by secreted IL6 and PGE2	Promote tumor growth and metastasis	(115)
BM-MSC	Gastric cancer	Induction of Treg by IL-15/STAT5 pathway	Increase tumor growth and metastasis	(116)
BM-MSCs	Head and neck squamous	Decrease of T cell proliferation by secreted IDO	Promote tumor growth and malignancy	(117)
BM-MSC	Breast cancer	Immunosuppression by secreted CXCL1, CXCL6, and CXCL8	Increased tumor growth and metastasis	(118)
GC-MSCs	Gastric cancer	Shift neutrophil toward promoting cancer by IL-6 through the STAT3-ERK1/2	Increased tumor growth gastric cancer	(119)
BM-MSC	Lymphoblastic leukemia	Inhibited P53 by secretion of PGE2-activated cAMP-PKA signaling	Support tumor growth of BCP-ALL	(120)
BM-MSC	Gastric cancer cells	TGF-β1 secreted by MSCs activated the SMAD2/3 pathway and supported cancer progression through the lncRNA MACC1-AS1/miR-145-5p/fatty acid oxidation (FAO) axis in cancer cells	Promoting gastric cancer progression	(121)
HCC-MSCs	Hepatocellular carcinoma	lncRNAMUF acted as a competing endogenous RNA for miR-34a, leading to Snail1 upregulation and EMT activation	Facilitates hepatocarcinogenesis	(122)
BM-MSC	Colorectal cancer cells	neuregulin 1 activated the HER2/HER3-dependent (PI3K)–AKT signalling pathway	Support tumor growth of colorectal cancer	(123)
LC-MSCs	Hepatocarcinoma	Induction of S100A4-miR155-SOCS1-MMP9 axis	Promote hepatocarcinoma progression	(124)
GA-hMSC	Glioblastoma	miR-1587 secreted in exosome- MSC reduces NCOR1	Support tumor growth of glioblastoma	(125)
LC-MSCs	Hepatocarcinoma	Expression of lncRNA *DNM3OS by MSC and effect on* DNM3OS/KDM6B/TIAM1 axis.	Cell proliferation and invasion *in vitro* and tumorigenesis and metastasis *in vivo*	(126)
BM-MSC	Gastric cancer	G6PD through the G6PD-NF-kB-HGF axis	Facilitated the progression of gastric cancer	(127)
CA-MSC	Ovarian tumor	increase BMP2, BMP4, and BMP6 in MSCs	Promotes tumorigenesis of ovarian tumor	(128)
CA-MSC	Breast cancer	promoted sphere formation via the EGF/EGFR/Akt pathway	Increased tumor growth breast cancer	(129)
hCC-MSCs	Colon cancer	microRNA (miR)-30a and miR-222 derived from colon CaMSCs through downstream target MIA3	Increased tumor growth. Proliferation and metastasis of colon cancer	(130)
BM-MSC	Colon cancer cells	miR-142-3p in exosomes stimulated the Notch signaling pathways through downregulating Numb	Increased the proliferation of colon cancer stem cells	(131)
BM-MSC	Breast cancer	secreted CCL5 by MSCs	Increasing the growth, motility, and metastasis of breast cancer cells	(132)
AD-MSC	Pancreatic cancer	Differentiating into CAFs	Promoting pancreatic cancer progression	(133)
BM-MSCs	Breast cancer	Neovascularization (secretion of VEGF, TGF-β, and IL-6)	Increased tumor growth	(134)
AD-MSCs	Breast cancer	AD-MSCs differentiated into cancer-associated myofibroblasts	Increased tumorigenesis and angiogenesis	(135)
AD-MSCs	Prostate cancer	periostin and TGF-β secreted by MSCs	Promoting the growth of Prostate cancer	(136)
BM-MSCs	Ovarian cancer	IL-6 by MSCs and transition to CAF	Promoting the growth of Ovarian cancer	(137)
AD-MSCs	Gastric tumor	MAPK pathway activation by MSC	Increased gastric tumor growth	(138)
BM-MSCs	Lung cancer	IL-6 by MSCs and regulated STAT3 signaling	Promoting the growth of lung cancer	(139)

MSCs properties and functions differ based on the activated receptor type. One of the most important of these receptors is Toll-like receptors (TLRs). Among these TLRs, if the TLR-3 receptor is active on the MSC, it is called MSC2 (TLR3-primed MSCs) (140). These cells (MSC2) have the property of suppressing immune cells by secreting anti-inflammatory cytokines such as IL1-RA and IL10. Subsequently, it promotes cancer cells. On the other hand, in the presence and activity of TLR-4 receptors on MSCs, they are called MSC1 (TLR4-primed MSCs). These cells (MSC1) secreted pro-inflammatory and pro-apoptotic factors such as IL17, GM-CSF, and TRAIL. MSC1 reduces the proliferation and inhibits the invasion of tumor cells (141).

It’s interesting to note that the TLR agonist exposure influences MSC function and aids MSCs in switching between MSC1 and MSC2 (anti-tumor or tumor growth promoter). In addition, MSCs can stimulate the growth of cancer cells and angiogenesis in different ways. For example, in prostate and breast tumors, MSCs increased pro-angiogenic factors such as VEGF MIP-2, IL-6, and TGF-β. These factors directly induce the proliferation of tumor cells and angiogenesis and thus increase the growth rate of solid tumors *in vitro* and *in vivo* (134). In addition, in a study on hepatocellular carcinoma (HCC), researchers discovered that the mRNA level of TGF-β1 was significantly increased. However, in the MSC-treated group, Smad7 mRNA expression was suppressed. Their research shows that MSCs may promote growth and angiogenesis via the TGF-1/Smad pathway (142). By producing chemokines, CA-MSCs increase the development and cell proliferation in cancer cells; for example, increasing the expression of CCL5 chemokine by BM-MSCs increases proliferation, migration, metastasis, and malignant behaviors in cancer cells (132). It has been found that CA-MSC forms a niche of cancer stem cells, which increases the ability of proliferation in cancer cells (143).

Regarding lymphoblastic leukemia, it has been found that PGE2 produced from MSCs activates the cAMP-PKA signaling pathway in tumor stem cells and inhibits the cancer suppressor function of p53, thereby increasing leukemogenesis (120). Also, it has been shown when MSCs are exposed to TME-like mediated oxidative stress, they can produce lactate, which will be absorbed by cancer cells, and result in producing ATP that will increase the growth and migration of cancer cells (144). Under TME circumstances, MSCs have been seen to differentiate into CAFs, stimulating cancer cell heterogeneity and playing an essential role in cancer progression. Another factor secreted by MSCs that directly affects the growth of tumor cells is neuregulin 1 (NRG1). NRG1 controls cell proliferation and differentiation via binding to EGFRs (145). *In vitro*, it has been determined that NRG1 produced by BM-MSCs activates the HER2/HER3-dependent PI3K-AKT signaling pathway in CRC and increases cell growth (123).

Other studies have reported that direct cell-cell interaction and co-culture between human UC-MSCs and MDA-MB-231 breast cancer cells can significantly increase the proliferation of tumor cells in mouse models (146), but its molecular mechanisms were not investigated. In a study, it has been determined that hepatocyte growth factor (HGF) secreted from BM-MSCs increases the expression of glucose-6-phosphate dehydrogenase (G6PD) in gastric cells and, subsequently, by affecting the G6PD/NF-κB/HGF axis in gastric cancer cells, increases glycolysis, proliferation, and metastasis of gastric cancer by upregulating c-Myc/HK2 signaling pathway (127). It has been suggested that another supportive function in tumor growth by MSCs is to increase cancer stem cell frequencies. This increase in ovarian cancer cells is due to the increased expression of bone morphogenetic protein (BMP)2, BMP4, and BMP6 factors on MSCs (128). Another method MSCs perform to support the growth of tumor cells is mammosphere formation. It has been shown in a study that when CA-MSCs are co-cultured with breast cancer cells, CA-MSCs induce mammosphere formation in these cancer cells using the EGF/EGFR/Akt pathway. As a result, it increases the growth of cancer cells (129).

Another tumor proliferation-enhancing effect of CA-MSCs is attributed to their role in protecting cancer cells from the immune system through the modulation of regulatory T cells and inhibition of NK cells, macrophages, and CTL functions (97). It has been found that gastric cancer-associated MSCs, by secreting IL-6 and IL-8 cytokines, as well as the JAK2/STAT3 signaling pathway, have caused the polarization of macrophages towards M2 macrophages, which promotes tumor growth (26). CA-MSCs in syngeneic melanoma mice (B16F10), by blocking cysteine in DCs via IL-10-STAT3 signaling, inhibited the proliferation of naive T cells and thus increased the growth and development of melanoma cancer cells (65). Also, by producing TGF-β1, CA-MSCs induce Tregs cells, which suppress immune responses in the breast tumor microenvironment, increasing the proliferation of these cancer cells (97). It has been found that the proximity of CA-MSCs isolated from ovarian cancer with active immune cells and the presence of ICP inhibitors destroy the response to treatment. This effect is caused by CCL2, CX3CL1, and TGF-β1 expression from CA-MSCs and the recruitment of CCR2^+^ monocytes and M2 macrophages in the TME, inhibiting TCD8^+^ cells (36). IDO is another substance secreted from CA-MSCs that suppresses anti-tumor immune responses. It has been reported that the cells in the head and neck squamous tumor area have decreased the proliferation and functions of CD4^+^ and CD8^+^ T cells against the tumor and increased the growth and invasion of these cancer cells (117).

MSCs decrease the ability of NK cells to secret IFN-γ, thus weakening their anti-cancer role and causing cancer cell growth (115). In addition, MSCs reduce the maturation of DCs and other APCs through PGE2 signaling; thus, T cells cannot be activated, and cancer cells continue to grow (147). Co-culture of MSCs with CD11b/Ly6G-positive neutrophils results in extensive inhibition of T cells *in vitro* and enhances breast carcinoma growth *in vivo* (148). It has also been reported that MSCs present in gastric cancer, by producing IL-6 through the STAT3-ERK1/2 signaling pathway, promote neutrophils and their shift towards the supportive phenotype of cancer cells (119). As a result, CA-MSCs secrete immune cell suppressor molecules and chemokines such as ICAM-I, PD-L1, VCAM, HLA-G, COX-2, IDO, TGF-β, PGE2, CXCL11, CXCL8, CXCL9, CXCL6, CXCL10, CXCL1, and promote cancer cells viability and increase their growth.

According to growing research, non-coding RNAs are involved in carcinogenesis, metastasis, and treatment resistance (149). Researchers demonstrated that human UC-MSCs strongly stimulate the growth of lung adenocarcinoma (LUAD) cells in a xenograft tumor model by transfecting miR-410 (150). A mouse cancer model showed that gastric cancer-derived MSCs could significantly increase the migration and growth of HGC-27 through increased miR-221 expression, which may act as a new biomarker in stomach cancer increase (151). Another study found that MSCs accelerated the development of gastric cancer by secreting TGF-β1, which triggered the SMAD2/3 pathway and the lncRNA MACC1-AS1/miR-145-5p/fatty acid oxidation (FAO) axis in cancer cells (121). In addition, in triple-negative breast cancer, MSCs strongly induce the regulation of RNA LINC01133 in adjacent tumor cells; this induction increases the spread of cancer stem cell-like phenotypic features and strengthens cancer cell growth (152). Also, it has been determined that in HCC-associated MSCs, a high expression level of lncRNA-MUF is observed; this lncRNA binds with Annexin A2 (ANXA2) and activates Wnt/b-catenin and causes their signaling and overexpression of miR-34a increases hence hepatocarcinogenesis (122). Microarray studies have shown that the expression of S100A4 on CA-MSCs is increased in liver cancer, and it has been determined that this molecule increases the growth of liver cancer cells by increasing the expression of mir-155 and finally by activating the STAT3 pathway and becomes proliferation of these cancer cells (124). In a study, it has been reported that when BM-MSCs enter the glioblastoma tumor environment, they become CA-MSCs, secrete mir-1587 into their exosomes in a specialized manner, and are absorbed by their cancer cells. mir-1587 reduces the level of NCOR1 in cancer cells and thus increases growth and proliferation in these cells (125). It has also been found that increasing the expression of DNM3OS lncRNA in CA-MSCs through the DNM3OS/KDM6B/TIAM1 pathway and interaction increases invasion, growth, and metastasis in hepatocarcinoma cancer cells (126). In breast cancer, it has been found that MSCs inhibit FoxP2 by increasing the expression of mir-214 and mir-199 in these cancer cells, thus increasing the growth, metastasis, and staying in the phenotypic state of cancer stem cells (153). Researchers have reported that high levels of miR-222 and miR-30a were found in the hCC-MSC secreted exosomes. These miRNAs, by targeting and reducing the expression of MIA3, increase the stimulation of growth, proliferation, and metastasis of colon cancer cells (130). On the other hand, it has been found that the increased expression of miR 221 in the exosomes obtained from CA-MCS cells of gastric cancer has increased the power of migration and tumorigenesis in these cancer cells (154). Additionally, it has been demonstrated that miR-142-3p, which is highly expressed in exosomes made from BM-MSCs, stimulates the Notch signaling pathway by decreasing the expression of Numb in cancer cells, which promotes the development and expansion of colon cancer stem cells (131).

In addition, CA-MSCs are able to differentiate into other tumor growth-supporting lineages. It has been shown that the treatment of BM-MSC with PC-3 prostate cancer cells supernatant increases their differentiation into the osteoblastic cell lineage. This process is done through the secretion of FGF-9 by PC-3 cells with the positive regulation of pro-osteoblastic factors, including fibronectin, integrin α5/β1, and osteoprotegerin compared to control environments, then support the growth of cancer cells (155). Finally, we can say that the mechanism of CA-MSCs is probably causing tumor cell proliferation by different factors, including angiogenic factors (βFGF, HIF-1α, and VEGF) (152); Chemokines (CCL5, CCL2, CXCL12, and CCL22) (156); growth factors (PDGF, SCF, HGF, IGF-1E, and GF) (157) and inflammatory cytokines (TGF-β, TNFα, IL-8, and IL-1β).

## 4 The role of CA-MSCs in tumor metastasis and invasion

As cancer cells alter their morphology, getting metastatic and invasive, they detach from the primary site and localize into a secondary organ far from the origin during metastasis (158). The interactions between cancer cells and other existing cells in the TME are necessary to generate a metastatic TME. One of these cells is CA-MSC, which migrates to tumor sites during inflammation, like the incidents during wound healing (159). The interaction between tumor cells and MSCs can be bidirectional. It has been demonstrated that carcinoma cells-derived IL-1 stimulates the production of PGE2, which in turn, in an autocrine manner with the cooperation of IL-1, induces the release of cytokines like IL-8 and IL-6 by MSCs, leading to the activation of Wnt/β-catenin signaling and stemness properties of cancer cells to enable tumor progression (25). In a model of breast cancer, it has been detected that cancer cells can stimulate the recruitment of BM-MSCs to tumor sites through the SDF-1α/CXCR4 axis. In turn, breast cancer cells metastasize to the bone marrow via SDF-1α, which belongs to the chemokine family and is known as a chemo-attractant mediator (160). In another study, it has been reported that the incubation of breast cancer cells with MSCs-derived exosomes elevates the migratory potential of cancer cells by inducing Wnt/β-catenin signaling. Indeed, the expression of Wnt/β-catenin targeted genes such as Axin2 and Dkk1 increases in exosome-treated cancer cells (161).

The active molecules produced by MSCs can contribute to generating an appropriate microenvironment for tumor metastasis. The role of IL-6 and IL-8 secreted by MSCs as inflammatory chemokines on tumor progression has been demonstrated in some cancer models (162, 163). Some tumor model studies have shown the role of inflammatory mediators derived from MSCs like CXCL1, CXCL2, or CXCL12 on metastatic and invasive properties of cancer cells through activating their specific receptors like CXCR2 and CXCR4 (164, 165). The MSCs-derived extrinsic factors can also induce the proliferation of cancer cells, potentially leading to distant metastasis. It is described that the released CXCL12 and IGF1 produced by MSCs activate the PI3K/AKT signaling pathway in breast cancer cells and then shift the cancer cell population towards more bone metastatic ones (166). The tumor-derived factors can induce the secretion of inflammatory chemokine and cytokines from CA-MSCs. For instance, it is revealed that osteopontin as a tumor-derived inflammatory cytokine promotes CA-MSCs to the secret high level of CCL5, which binds to its receptor (CCR5) on cancer cells to enhance cancer cells’ metastatic potential and MSCs’ ability to migrate to metastasis site (132, 167, 168). In prostate cancer, it is shown that CCL5-derived from MSCs and cancer cells, through suppressing the nuclear translocation of androgen receptors, increases the metastatic potential of cancer cells due to inhibiting androgen receptor signaling (169). It is indicated that blocking these receptors with neutralizing antibodies can repress tumor metastasis in a mouse model of breast cancer (170). Indeed, it is identified that CA-MSCs secret high levels of asporin into tumor stroma, promoting metastatic tumor development and restricting MSCs differentiation through binding to BMP-4 (171).

Furthermore, in a model of breast cancer, it is documented that MSCs induce the metastases and invasive behavior of breast tumors by altering the cancer cells gene expression profiles by upregulating the oncogenic pathways like Wnt and TGF-β and thereby, enhancing the expression of genes related to the cell membrane and matrix-associated proteins (172). Another study showed that MSCs, via upregulating the miR-199 and miR-214, inhibit the expression of the FOXP2 transcription factor in cancer cells to stimulate breast cancer metastasis (153). Moreover, in human MSCs-treated hepatocellular tissues, TNF-α, IL-6, and α5 integrin expression were elevated to promote tumor growth and metastasis in HCC (173). Enhancement in the migratory and invasive ability of glioblastoma cells has also been correlated to TGF-β secreted by MSCs (174). TGF-β, as a growth factor, is implicated in generating invasive and metastatic properties of cancer cells by inducing epithelial-to-mesenchymal transition (EMT) (175). In a co-cultured model of breast cancer cells with MSCs, it has been reported that the migratory capacity of cancer cells increases through the ER-SDF-1/CXCR4 pathway (164). In another study, the role of BM-MSCs in activating the migratory capacity of breast cancer cells has been attributed to the CXCR2 receptor (165). In a mice model of gastric cancer, CA-MSCs enhances the survival and migration of cancer cells by upregulating the expression miR-221 (151). It has been documented that MSCs could transmit mitochondria to glioblastoma stem cells and breast cancer cells to enhance the proliferative and invasive potency of cancer cells through increasing oxidative phosphorylation (OXPHOS) and adenosine triphosphate (ATP) production (176–178).

Several studies declare the crucial role of MSCs in EMT, a process by which cancer cells obtain the stem cell phenotype to migrate to other sites and metastasis. A study on pancreatic cancer has indicated that human BM-MSCs promote EMT and cancer-initiating stem cell-like characteristics in cancer cells via the Notch signaling pathway (179). AT-MSCs have been verified to upregulate the expression of EMT-related genes in invasive breast cancer cells through TGF-β and expressing BMP (180).

Some studies describe the role of MSCs in accelerating the metastasis of tumors by altering the expression of enzymes related to metastasis. It has been shown that Lysyl oxidase is upregulated in breast cancer by recruiting MSCs to modulate breast cancer invasion, metastasis, and EMT (181). Moreover, exosomes released from AT-MSCs have been stated to enhance the expression of homosapien collagen beta galactosyl transferase 2 (COLGALT2), which is a crucial enzyme for collagen glycosylation, to promote metastasis and tumor proliferation in osteosarcoma cells (182). Also, it has been demonstrated that MSCs-derived exosomes enhance the tumorigenic features of ovarian and breast cancer cells through upregulating MMP-2 and MSCs-related markers such as CD73 and CD90 (183). MMPs are crucial for the degradation of ECM (184), and MSCs, by secreting MMPs, play an important role in inducing a pro-metastatic environment (185). Some MSCs-secreted factors, such as TGF-β, can also stimulate tumor fibrosis (186), which can cause the retention of chemokines and growth factors in the fibrotic environment to accelerate metastatic growth (187). In addition, MSCs can cooperate in downregulating or degrading E-cadherin in tumor cells; E-cadherin acts as an adhesion protein to inhibit cancer cell dissociation (188).

The role of MSCs in preparing pre-metastatic sites for circulating cancer cells is shown in some evidence (189–191). It is determined that CA-MSCs express receptors for VEGF, which not only enhance the migration of MSCs to tumor site (192) but also, with high levels of CXCL12 and deposited fibronectin, increases the migration and adherence of lung carcinoma and melanoma cells to pre-metastatic niche (193). Cancer-educated BM-MSCs, as a TME component, induce lung cancer cells’ survival at primary and metastatic sites by extending BM-PMN-MDSCs during the metastasis (194).

By contrast, the inverse role of MSCs on tumors has also been reported in various studies (195–197). In an HCC model, human MSCs suppressed the metastatic potential of cancer cells via downregulating TGF-β despite enhancing tumor growth (198). Recently, it has been indicated that MSCs-derived exosomes convey miR-3940-5p, which reduces the metastasis of colorectal cancer cells by targeting the α6 integrin family and then inactivating TGF-β1 (199).

The recruited MSCs in the TME can get the CAFs-phenotype to contribute to TME in cancer progression and chemoresistance (200). The initial *in vitro* evidence in this field has found that treatment of BM-MSCs with conditioned media of human breast cancer, pancreatic cancer, and glioma lead to the differentiation and expression of CAFs markers like α-SMA, fibroblast associated protein (FAP), fibroblast-specific protein 1 (FSP1) and vimentin (201). The CA-MSCs express lower amounts of vimentin and FSP1 in comparison with CAFs (202). The excessive capacity of CA-MSCs to differentiate into CAFs versus typical MSCs has been demonstrated in multiple studies (12, 203). An animal model of inflammation-promoted gastric cancer has discovered that more than 20% of CAFs originate from BM-MSCs, which can amplify during tumor progression and induce the probability of malignancy (202). Numerous secreted factors to the TME trigger the signaling pathways to stimulate the CA-MSCs differentiation to CAFs. It is indicated that the secreted factors by tumors activate the TGF-β/Smad signaling pathways in CA-MSCs to facilitate their differentiation to the CAF phenotype (204, 205). In gastric cancer, it is observed that the migrated MSCs to the inflammation site of tumor stroma generate CAFs through activating TGF-β and SDF-1α pathways by TGF-β and CXCL12 isolated from cancer cells and the TME (202) or by tumor derived exosomes (206). It seems that TGF-β plays a significant role in attracting and recruiting MSCs to tumor stroma.

On the other hand, TGF-β secreted by cancer cells and tumor stroma is crucial in the transition of MSCs into CAFs-like cells, which in turn promotes tumor progression (204). Monitoring the fate of MSCs during tumor progression would help better distinguish the heterogeneity of MSCs in the TME and consider its role in therapeutic strategies. Additionally, MSCs are involved in the progression of cancer cells by releasing active molecules such as SDF-1, CCL5/CCR5, CCR2, TNF-α, TGF-β, etc. (**Table 4**). Therefore, novel therapeutic approaches are needed to suppress or mediate the interaction between cancer cells and MSCs.

**Table 4:** The role of CA-MSCs in increasing tumor metastasis by different mechanisms.

MSCs source	Cancer type	Result	Molecular Mechanism	Reference
Human BM-MSCs	Breast cancer cells	Activate metastasis	By upregulating lysyl oxidase production	(181)
BM-MSCs	Breast cancer cells and mice	MSC facilitates breast cancer cells’ entry into bone marrow	Through Tac1-mediated regulation SDF-1α)/CXCR4 axis	(160)
BM-MSCs	Prostate cancer	Enhance prostate cancer cells’ metastatic capability and cancer stem cell population	Through secreting CCL5 to inhibit androgen receptor signaling	(169)
Human BM-MSCs	Human carcinoma cell lines and nude mice	Promote stem cell–like phenotype in carcinoma cells	PGE2 and IL-1 induce the production of IL-8 and IL-6 by MSCs	(25)
VEGFR1-positive hematopoietic bone marrow progenitors	Melanoma cells and lung carcinoma cells	Prepare an appropriate environment for colonization of circulating cancer cells	By Expressing VEGFR and with the contribution of CXCL12 and fibronectin	(193)
Human BM-MSCs	Hepatocellular carcinoma	Induces tumor growth and metastasis	Via MAPK pathway and enhancing the expression of TNF-α, IL-6, and integrin α5	(173)
Human BM-MSCs	Breast cancer cells	Activate metastasis	By stimulating the hypoxia-inducible factors (HIFs)	(207)
Human adipose stromal cells	Breast cancer cells	Enhance migration and invasion	By producing IL-6	(208)
Human BM-MSCs	Breast carcinoma spheroids	Increase metastasis	with degradation of E-cadherin via activating ADAM10	(188)
MSC derived from different human tissues	Glioblastoma	Elevate the migratory and invasive potency of glioblastoma cells	By producing TGF-β1	(174)

## 5 The role of CA-MSCs in tumor chemoresistance

CA-MSCs are one of the crucial actors in chemotherapy resistance, and they point to potential targets to improve patients’ response to chemotherapy. According to the research of Jeanine M.L. Rood Hart, endogenous MSCs become activated when treated with platinum analogs and release substances that protect tumor cells from a variety of chemotherapeutic agents. Two different polyunsaturated fatty acids caused by platinum (PIFAs), 12-oxo-5,8,10 hepta decatrienoic acid (KHT) and hexadeca 4,7,10,13 tetraenoic acid [16:4(n3)] were discovered using a metabolomics approach. These PIFAs are minute amounts that can result in resistance to various chemotherapeutic drugs. Surprisingly, MSC-induced resistance can be prevented by inhibiting the key enzymes [thromboxane synthase and cyclooxygenase-1(COX-1)] that produce these PIFAs (209). The study examined this possibility to understand the underlying molecular mechanism and determine if exosomes produced from MSCs mediate gastric cancer chemotherapy resistance. They discovered that MSCs-derived exosomes dramatically induced 5-fluorouracil (5-FU) resistance to gastric cancer cells. MSCs-derived exosomes increased the expression of multi-drug resistance-related proteins, such as MDR, MRP, and LRP, and inhibited the 5-FU-induced apoptosis. In gastric cancer cells, MSCs derived exosomes functioned mechanistically to activate the Raf/MEK/ERK kinase cascade and calcium/calmodulin-dependent protein kinases (CaM-Ks). The boosting function of MSCs-derived exosomes in chemoresistance was decreased by hampering the CaM-Ks/Raf/MEK/ERK pathway. Inducing drug resistance in gastric cancer cells through activating the CaM-Ks/Raf/MEK/ERK pathway is one potential effect of MSCs-derived exosomes. Chemotherapy for gastric cancer may be more effective if it focuses on how MSCs-derived exosomes interact with cancer cells (210).

HCC has an important trait of inflammation. A few inflammatory cytokines released in the TME alter how MSCs operate. In both *in vivo* and *in vitro* settings, they noticed that MSCs pretreated with the cocktail of IFN-γ and TNF-α promoted resistance to treatment in HCC cell lines. HCC cell line cells experienced autophagy after exposure to MSCs pre-treated with IFN-γ and TNF-α. The HCC cells use this process as a defensive mechanism to tolerate the cell toxicity of chemotherapy medicines. Treatment of the HCC cells with an autophagy inhibitor successfully decreased the MSCs-induced resistance to chemotherapy in these cells. TNF-α and IFN-γ-induced stimulation in MSCs induced TGF-β expressions (211). Another pathway by which CA-MSCs can cause drug resistance in tumor cells is the WNT signaling pathway (212). Studies show that the co-culture of acute lymphoblastic leukemia (ALL) cells and MSCs can lead to the reduction of apoptosis caused by cytarabine in tumor cells (213). The use of Wnt signaling inhibitor agent increased the sensitivity of ALL cells to chemotherapy and compensated for the inhibition of apoptosis induced by MSCs (213). In addition, it has been shown that CA-MSCs can activate the expression level of sphingosine-1-phosphate receptor 1 (S1PR1) in neuroblastoma-related tumor cells and the downstream signaling pathway that functions through JAK2 and STAT3 (214). S1PR1 overexpression protects cancer cells from chemotherapy-induced apoptosis by activating JAK-STAT3 signaling (215). Co-culture of MSCs by oral squamous cell carcinoma (OSCC) also leads to the drug resistance of cancer cells to cisplatin through the activation of the signaling pathway related to PDGFR-α/AKT (216, 217). Studies conducted by Bing Tu et al. show that the co-culture of MSCs with osteosarcoma cells leads to increased resistance to doxorubicin or cisplatin in tumor cells through a STAT3-dependent pathway (218). It has also been determined that pretreatment of MSCs with IL-6 increases their ability to induce STAT3 signaling pathway and induce drug resistance (218).

Another way MSCs contribute to the drug resistance of tumor cells is the acceptance of damaged mitochondria from tumor cells by tunneling nanotubules (TNTs) (219). Inhibiting the formation of TNTs by cytochalasin D leads to a decrease in the transfer of damaged mitochondria from tumor cells to MSCs and leads to a reduction in cancer cells’ drug resistance (178, 220). It also seems that MSCs can transfer healthy mitochondria to tumor cells through these TNTs and induce drug resistance in them.

Aldehyde dehydrogenase (ALDH) activity has a direct relationship with the drug resistance of cancer cells (221). Different studies have shown that MSCs increase the ALDH activity in cancer cells by the production of TGF-β through the p38-dependent signaling pathway. Inhibiting or reducing the production of TGF-β from MSCs decreases their ability to induce drug resistance in tumor cells (222).

Glioma-associated MSCs consist of two distinguishable populations characterized by the level of CD90 expression (223). CA-MSCs that express CD90 at a high level play a role in the proliferation, differentiation, adhesion, and migration of glioma tumor cells and have no effect on drug resistance induced in tumor cells. However, CA-MSCs CD90^low^ are of great importance in the drug resistance of tumor cells. These cells, by increasing the expression of FOXS1 in glioma cells, lead to a decrease in the sensitivity of tumor cells to the drug temozolomide and apoptosis in these cells (224).

## 7 The role of CA-MSCs in tumor angiogenesis

Carcinoma-associated mesenchymal stromal/stem cells in the TME can affect the progression of ovarian tumors by promoting angiogenesis at the tumor site, which speeds up tumor growth. It has been shown that endothelial cells, CA-MSCs, and ovarian adenocarcinoma cells secrete pro-angiogenic cytokines, including IL-6, IL-8, and VEGF, at higher levels when they are in contact with a particular cell type (225). CA-MSCscan also induces macrophage differentiation to an M2 phenotype and activates them, causing them to release a large amount of pro-angiogenic cytokines that are helpful for the advancement of all related ovarian cancer cells that have been studied (226). According to research by W-H Huang et al., the angiogenesis and tumor growth rate are both accelerated when different CRCs are combined with non-tumorigenic MSCs. IL-6 released from MSCs stimulates the release of endothelin-1 (ET-1) in cancer cells, which in turn triggers the activation of Akt and ERK in endothelial cells, improving their ability to attract other cells to the tumor and promoting angiogenesis (227). An anti-IL-6 antibody or lentiviral-mediated RNAi against IL-6 in MSCs, the inhibition or knockdown of ET-1 in cancer cells, or the suppression of ERK and Akt in host endothelium cells can all be used to target the IL-6/ET-1/Akt or ERK pathway of tumor-stroma interaction. These show that efforts to stop the interaction between MSCs and cancer cells aid in preventing angiogenesis and reducing tumor growth. These findings indicate that targeting the interaction between the proangiogenic factors secreted by cancer cells and the tumor microenvironment, specifically the IL-6 released by MSCs, may result in novel therapeutic and preventive approaches (225).

MSCs transplanted into mice support tumor angiogenesis *in vivo* through the expression and production of VEGF and lead to an increase in the density of CD31^+^ vessels after MSC transfer. The use of siRNAs that interfere with VEGF expression reduces the ability of MSCs to induce angiogenesis at the tumor site (228). It has also been shown that MSCs bind to blood vessel endothelial cells after intratumoral injection and express procytic markers such as αSMA, NG2, and PDGFRβ. Pericytes promote tumor growth and tumor-related angiogenesis through the production of various proangiogenic soluble factors (229).

## 7 Conclusion and future perspective

**Figure 2:**
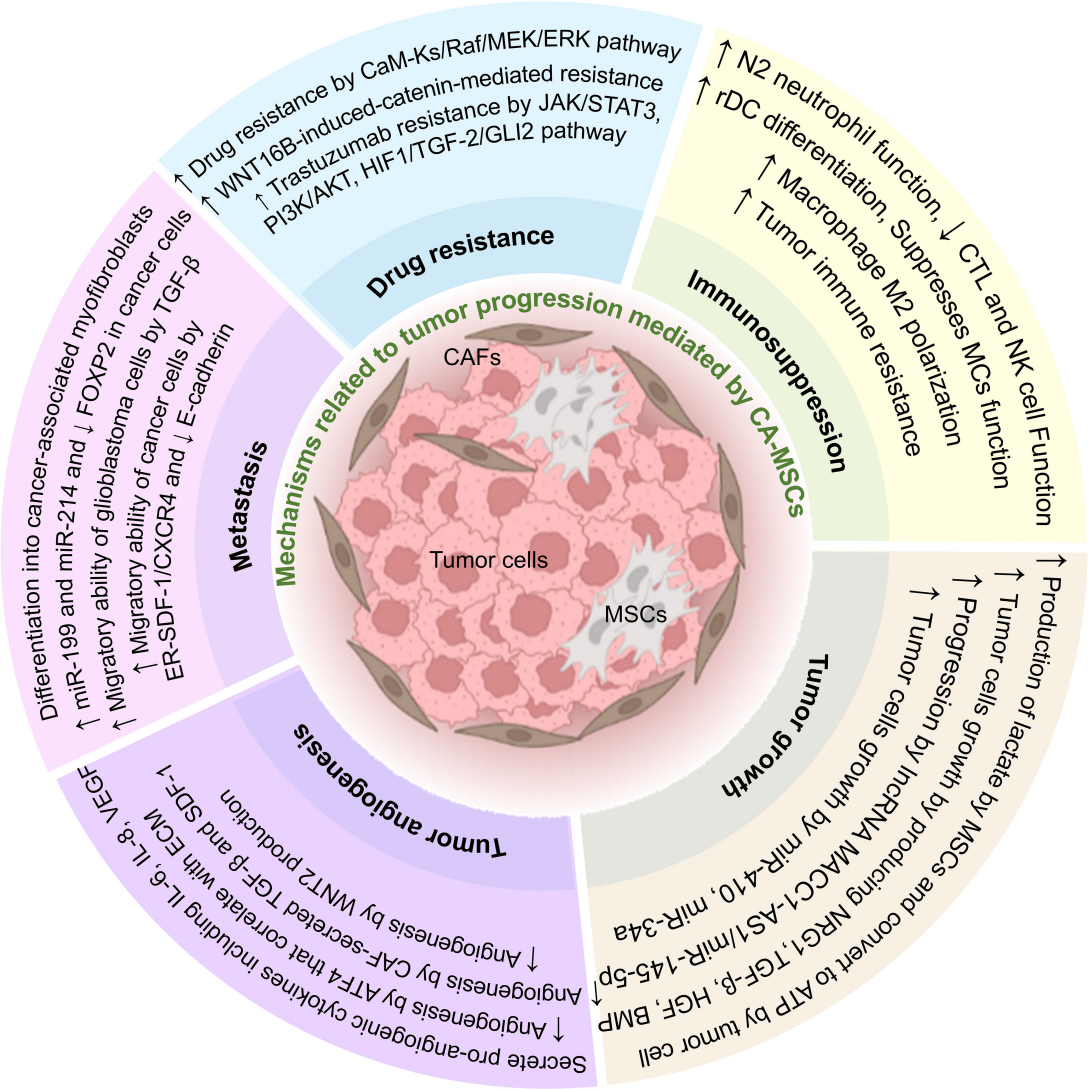
The effect of CA-MSCs on tumor progression. CA-MSCs contribute to tumor growth by affecting the main mechanisms of tumor expansion, including development, angiogenesis, invasion, metastasis, and drug resistance. Also, these cells contribute to the expansion of the tumor by suppressing the responses related to the immune system in the tumor microenvironment.

As mentioned in this article, intercellular communication in TME can affect most aspects of tumor development (**Figure 2**). One of the most important cells that affect tumor cells is MSCs, which can also differentiate into fibroblasts (CAFs). Due to their characteristics, these cells can increase the expansion and production of cancer stem cells and help the stability of the tumor.

Also, CA-MSCs can contribute to tumor progression by suppressing the immune system, increasing metastasis, invasion, angiogenesis, tumor growth, and survival of tumor cells, and also by increasing drug resistance in tumor cells. In addition, MSCs can differentiate in the tumor environment and change the conditions of the TME in favor of the tumor. Intercellular communication in TME is very complex, and CA-MSCs usually exert their effect on tumor cells by using three mechanisms, including producing extracellular vesicles, soluble mediators, and direct cell-to-cell contact. According to the mentioned studies in this article, it seems that CA-MSCs are one of the main culprits of tumor expansion. So, it can be concluded that preventing the spread of CA-MSCs can also prevent the spread of tumors. It is very important to know this point because cancer stem cells are troublesome in many cancers and can lead to tumor recurrence after treatment. Also, preventing the spread of CA-MSCs can partially prevent metastasis, invasion, and drug resistance induced by these cells in tumor treatment.

On the other hand, inhibiting the expansion and function of CA-MSCs can lead to removing the suppression of the immune system against tumor cells and decrease the tumor’s power in immune evasion so that functional immune responses against tumor expansion are formed. As mentioned, MSCs, by expressing chemokine receptors related to migration to the site of inflammation, in clinical uses, they may migrate to the site of chronic inflammation induced by the tumor. According to the authors, they suggest the use of engineered MSCs that do not express these chemokine receptors (230). However, the lack of chemokine receptor expression can affect MSCs’ therapeutic potential and their migration to the target site. This issue can be solved through local injection of MSCs. Until now (September 2023), this approach has not been used in any of the pre-clinical or clinical studies. For this reason, the authors of this article, considering the importance of the presence of CA-MSCs in the tumor tissue and their role, encourage researchers to investigate this matter. However, as mentioned, TME is very complex, and proving the role and therapeutic importance of removing or preventing the spread of CA-MSCs requires more studies.

## Author contributions

AH: Conceptualization, Methodology, Supervision, Visualization, Writing – original draft, Writing – review & editing. KM: Investigation, Writing – original draft, Writing – review & editing. ZM: Writing – original draft, Writing – review & editing. AK: Writing – original draft, Writing – review & editing. NR: Writing – original draft, Writing – review & editing. NH: Writing – original draft, Writing – review & editing. AA: Writing – original draft, Writing – review & editing. SS: Conceptualization, Supervision, Writing – review & editing.

## Glossary

**Table:**

(MSC)	Mesenchymal stem/stromal cells
(CA-MSCs)	Cancer-associated mesenchymal stem/stromal cells
(BM-MSC)	Bone marrow-derived mesenchymal stromal/stem cell
(UC-MSCs)	Umbilical cord-derived mesenchymal stem/stromal cells
(TME)	Tumor microenvironment
(EMT)	Epithelial-to-mesenchymal transition
(TAMs)	Tumor-associated macrophages
(CAFs)	Cancer-associated fibroblasts
(iCAFs)	Inflammatory CAF
(myCAFs)	Myofibroblastic CAFs
(CSCs)	Cancer stem cells
(CAAs)	Cancer-associated adipocytes
(MDSCs)	Myeloid-derived suppressor cells
(LPS)	lipopolysaccharide
(TNF-α)	Tumor necrosis factor-α
(ECM)	Extracellular matrix
(ROS)	Reactive oxygen spices
(HIF)	Hypoxia-induced factor
(MCP-1)	Monocyte chemotactic protein-1
(MIP-1α)	Macrophage inflammatory protein-1 alpha
(TANs)	Tumor-associated neutrophils
(GC-MSCs)	Gastric cancer mesenchymal stromal/stem cells
(NKs)	Natural killer cells
(PGE2)	Prostaglandin E2
(IDO)	Indoleamine 2,3-dioxygenase
(DCs)	Dendritic cells
(rDCs)	Regulatory DCs
(MCs)	Mast cells
(VEGFs)	Vascular endothelial growth factors
(CSF-1)	Colony stimulating factor-1
(MMP)	Matrix metalloproteinase
(CTLs)	Cytotoxic T lymphocytes
(Th)	Helper T
(TGFB1)	Transforming growth factor β1
(iNOS)	Inducing nitric oxide synthetase
(TLRs)	Toll-like receptors
(NRG1)	Neuregulin 1
(PI3K)	Phosphoinositide 3 kinase
(BMP)	Bone morphogenetic protein
(α-SMA)	Smooth muscle alpha-actin
(5-FU)	5-fluorouracil
(CaM-Ks)	Calcium/calmodulin-dependent protein kinases
(FAP)	Fibroblast activation protein
(GC)	Gastric cancer
(NSCLCs)	Non-small cell lung malignancies
(ANXA2)	Annexin A2
(CRC)	Colorectal cancer
(ECs)	Endothelial cells
